# Introduction: Embracing Ambivalence and Change**

**DOI:** 10.1002/bewi.202200044

**Published:** 2022-09-09

**Authors:** Lara Keuck, Kärin Nickelsen

**Affiliations:** ^1^ History and Philosophy of Medicine Bielefeld University; ^2^ Max Planck Institute for the History of Science Berlin Germany; ^3^ History of Science LMU Munich Germany

## 1 Beginnings

In 1997, Hans‐Jörg Rheinberger published his now seminal book *Toward a History of Epistemic Things: Synthesizing Proteins in the Test Tube*. Twenty‐four years later, in 2021, he compiled a collection of essays under the title *Spalt und Fuge: Eine Phänomenologie des Experiments*, which will shortly also be available in English. What happened between these two books? What does it mean to write the history of the life sciences now? What is the place of Rheinberger's historical epistemology in the contemporary landscape?

These were the questions that we, the editors, started discussing in the summer of 2021. The occasion was not only Rheinberger's latest book, but also the more mundane fact that one of us, Lara Keuck, had just joined the editorial team of this journal. The other one of us, Editor‐in‐Chief Kärin Nickelsen, therefore proposed to collaboratively edit a small topical collection, dedicated to their mutual interest in the history and historiography of the life sciences, in order to introduce the novice to the inner workings of journal making. Rheinberger's *Spalt und Fuge* would loosely serve as a starting point for a forum of four or five short contributions, mainly from early and mid‐career scholars in the field. The project would avoid any *Festschrift* character (since several of them had been published already^1^); instead, we wanted to initiate a discussion about how topics and concepts associated with Rheinberger's work, and others that originated in the same period, are dealt with today. After all, we are now starting to write the history of life sciences during the 1990s, when some of our favorite historiographical tools were invented. What does this mean for our distinction between actors’ categories and analytical categories? Are concepts such as the *experimental system* still helpful, given the enormous changes within both the life sciences and their historiography? We drafted a one‐page concept paper and started to send out invitations.

**Figure 1:**
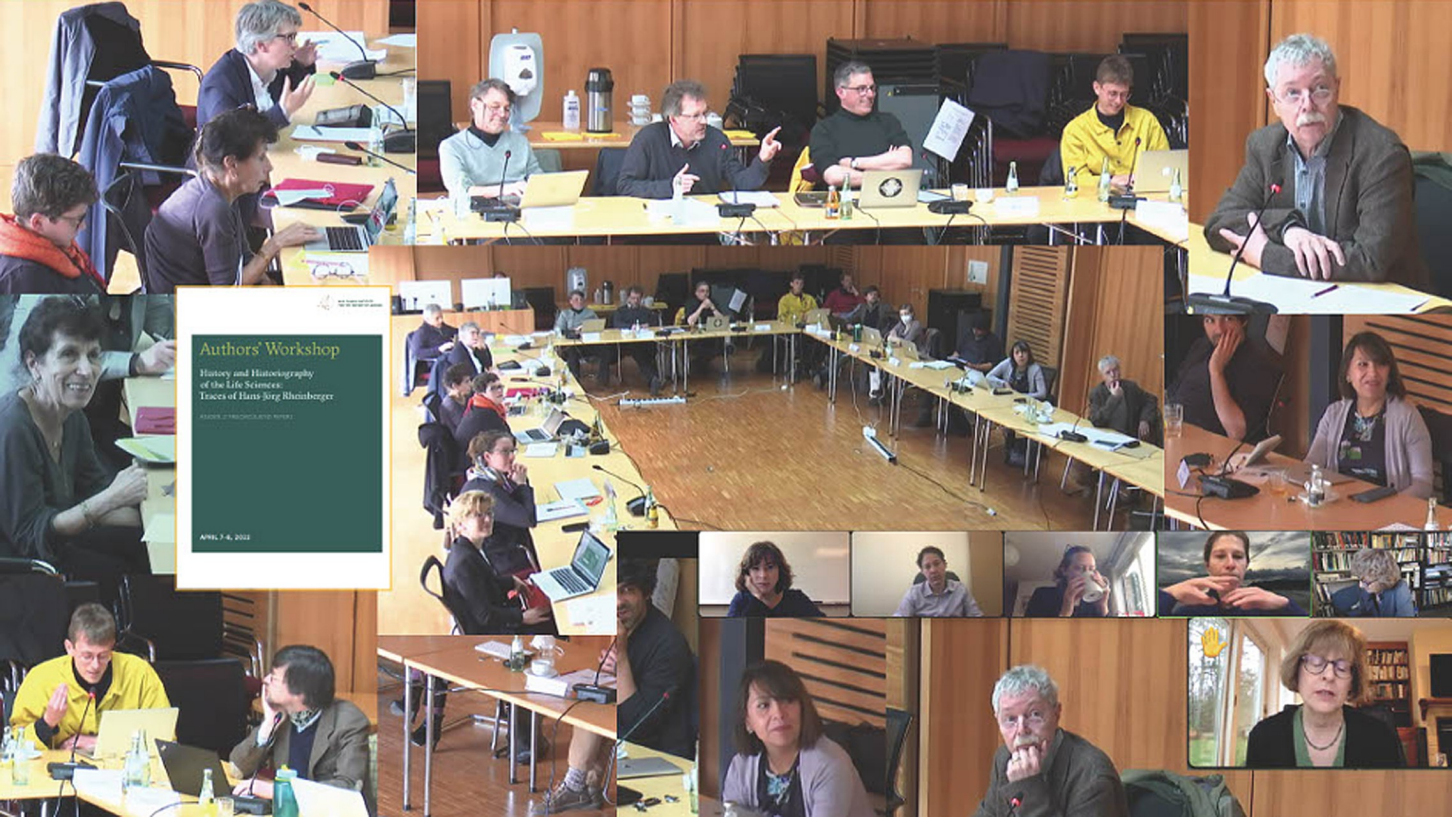
Collage of workshop impressions, 7–8 April 2022, Berlin and online. Photographs and arrangement: Caterina Schürch.

The project developed a dynamic that we had not anticipated. Our colleagues thought the questions were timely and worthwhile; however, they also inquired about the scope of our collection and the invitees. We realized that we needed to include more voices, from scholars across academic generations with different degrees of proximity to Department III of the Max Planck Institute for the History of Science (MPIWG) under Hans‐Jörg Rheinberger's directorship (1997–2011). Thus, in between recurrent waves of the COVID‐19 pandemic, we asked some of the busiest scholars in our field to write an essay within a ridiculously short timeframe—and, miraculously, they agreed. In early April 2022, we met in person and on screen, for an authors’ workshop at the MPIWG (Figure 1). We had, meanwhile, added a subtitle to our initial proposal, which read *Traces of Hans‐Jörg Rheinberger*. We deliberately chose the Rheinbergian term *traces* because, despite our broad invitation, all of the essays sought connection in some way or another to Rheinberger's work. Moreover, the memories of engaging with *Epistemic Things* and being part of the community in and around Dept. III clearly played an important role in the amazing turnout. We still were unwilling to add another *Festschrift* to the list, but we acknowledged the paradox of the project in the title of our introductory remarks, *Ceci n'est pas un hommage*.

## 2 Conjunctures, Traces, Fragments

The workshop was a remarkable hybrid of events. At times, the colloquium of Department III was revived (through the participation of Rheinberger and long‐standing members such as Christina Brandt and Staffan Müller‐Wille), and at other times it was historicized. For those who had never belonged to Rheinberger's department, including the editors, it was an exciting experience. For others, it had the bittersweet flavor of nostalgia with a pinch of deconstruction. At the end of the workshop, blessed (and challenged) with a colorful bouquet of presentations, we suggested three Rheinbergian terms that we would use to cluster the papers for their publication: *Conjunctures*, *Traces*, and *Fragments*. In his *Postscriptum* to this collection, Hans‐Jörg Rheinberger himself reflects on the meaning of these categories in his work, and we happily refer to this essay for an illuminating introduction into his intellectual cosmos.

### 2.1 Conjunctures

The essays that we assigned to the first section, ***Conjunctures***, all deal in one way or another with different roles of experiments, and the question of how to analyze them in ways that are epistemologically fruitful and historically sound.

**Michel Morange** starts with a historiographical as well as personal retrospective of Rheinberger's *Toward a History of Epistemic Things*. Morange emphasizes its exceptional originality at the time, in particular the masterful balance between reconstruction and deconstruction. One of the most important achievements of the book, Morange holds, was the replacement of experience, which stresses the role of individual protagonists, to the momentum of the *experimental system*. While this has prompted valuable studies, Morange reminds us “there remains much work ahead to take advantage of its full potential.”

**Caterina Schürch**, in contrast, offers an alternative framework for analyzing experimental work. Complementary to typical Rheinbergian interests in structures, series, and transformations, Schürch focuses on the protagonists’ goals, norms, resources, and skills. The elegant analysis of two examples from the first half of the twentieth century demonstrates how this approach reveals layers of experimentation that otherwise remain hidden: “Experiments,” Schürch claims, “have the potential to integrate disciplines; not only the physical and biological sciences, but also the historiography, philosophy, and sociology of the life sciences.”

**Kathryn Maxson‐Jones** then highlights the equally intriguing potential of experiments to prompt changes within *one* discipline. In her account of the extraordinary career of the squid giant axon in twentieth‐century neurobiology, Maxson‐Jones picks up Rheinberger's concept of *epistemic thing* and develops it further. The paper shows how the comparison of the squid axon with other neurons changed what figured as an *epistemic thing* in neurobiology; and while doing so, Maxson‐Jones *en passant,* and similar to Rheinberger, turns the actors’ category of “preparations” into an analytical one.

**Edna Suárez‐Díaz** points to the importance of more embedded analyses of experimental research. She uses the case of electrophoresis in postwar Mexico to argue for an integration of Rheinberger's concepts of *technical* and *epistemic things* as well as *experimental systems* with perspectives from the social history of technology. Only then, Suárez‐Díaz claims, can we begin to understand how cultures of experimentation emerge within commercial, industrial, and colonial relations of power.^2^

**Hanna Worliczek** combines Rheinberger's *experimental cultures* and the analysis of the epistemic mainstreaming of disciplines. Worliczek argues that post‐1960 cell biology was a heterogeneous complex of competing experimental sub‐cultures, scholarly practices, and epistemic virtues. Worliczek traces how in this context “descriptive” came to be used as a dismissive label; while in reaction, this type of work was increasingly framed in a different terminology. One of the effects was that the historiography of later periods emphasized the experimental nature of the field, despite the fact that careful descriptions continued to be a crucially important part of cell biologists’ work.

**Alexander von Schwerin** reminds us how important the circulation of technology, materials, and personnel has been in molecular biology. The case of Brigitte Wittmann‐Liebold's research at the Max Planck Institute for Molecular Genetics highlights some of the non‐epistemic factors that influence circulation, including organizational frameworks, economics, and gender. These three factors prevented Wittmann‐Liebold from having the career she deserved and in doing so kept her *experimental system* from reaching its full potential. In view of this episode, the paper calls for a more inclusive view of experimental cultures and circulation, which also considers socio‐political and economic conditions.

**Robert Meunier**, finally, takes us into the world of post‐experimental research. Precision medicine, and other areas of data‐intensive work, arguably can no longer be analyzed in terms of *experimental systems*. Epistemic goals, practices, and methodology of this research differ dramatically from twentieth‐century laboratory work, and consequently require a different set of analytical categories. Meunier introduces the category of “approach” as a more encompassing alternative, and adds an illuminating reflection on how and when the concepts and categories of science studies (in a broad sense) become outdated, if their subject of inquiry changes.

### 2.2 Traces

The second cluster of papers deals with the ***Traces*** in and of Rheinberger's work. They are in part first attempts to historicize historical epistemology, and in part highlight Rheinberger's contributions in new ways.

**Christian Reiß** sketches the history of a German‐speaking tradition of biological philosophy that emerged in the late nineteenth century and unfolded during the first third of the twentieth. Reiß distinguishes three philosophical strands, neo‐Kantianism, phenomenology, and *Lebensphilosophie*, all of which developed vitalist and holistic conceptions of life. Contrary to widespread assumptions, this line of thought continued to be influential in Germany after 1945, although it became more and more detached from the (molecular) biological research at the time. As Reiß claims, even in Rheinberger's work, “we can see the *‘Spalten*’ and ‘*Fugen*’—the continuities and discontinuities—that this tradition left there.”

Reiß’s paper is nicely complemented by **Pierre‐Olivier Méthot's** contribution, who juxtaposes Rheinberger's œuvre with the contemporary Anglo‐American philosophy of biology. Rheinberger's interest in practices instead of theories, and his focus on molecular instead of evolutionary biology made it difficult to find common ground. In fact, Méthot points out that “Rheinberger never wanted to be considered as a philosopher of biology, but primarily as an ‘epistemologist,’” in the French tradition. This diagnosis of intellectual marginality is balanced, however, with the observation of Rheinberger's productive legacy in the present.

**Cornelius Borck** analyses the role of the tactile and the visual in Hans‐Jörg Rheinberger's œuvre, and he points to the central role that Ludwik Fleck's *Widerstandsaviso* has played in Rheinberger's work. Borck argues, in contemporary data‐driven approaches, that the materiality, and thereby the *Widerstandsaviso*, of an *epistemic thing* recedes behind visualizations and abstractions. Borck traces how this shift is reflected in Rheinberger's more recent work. The essay can also be read as an invitation to reassess our readings of Rheinberger, Bachelard, and Fleck: they no longer reflect how knowledge *is being* generated, but rather an epistemology that has increasingly become marginalized and outdated—and hence epitomizes what is lost in data‐driven science.

**Stephen Hilgartner** enriches our collection by insightful comments from the perspective of Science and Technology Studies (STS). When we invited Hilgartner to contribute to our project, we suggested, tongue‐in‐cheek, that he commented on the fact that historians are always late to the party, since STS often works on a topic for a long time before historians discover it. Hilgartner turns this into a serious methodological question about the temporality of science studies. He explores the potential superiority of ethnographic or archival research, and concludes that the dichotomy is specious. Since both are subject to recursive contingency of interpretation, we, the editors, believe that we clearly should celebrate all parties together.

**Michael Zimmermann** provides a thoughtful contextualization of Rheinberger's work, speaking as a longtime colleague and friend from a neighboring discipline, namely, art history. The essay helps to understand the influences of continental philosophy and art history on Rheinberger's thinking about non‐linear temporality, while it also shows how Rheinberger's reflections on historical epistemology became so influential in various other disciplines. Rheinberger's epistemic times, we learn in Zimmermann's essay, have a history, present, and future far beyond the history and philosophy of the life sciences.

**Elisabetta Mengaldo** takes us into one of these realms where Rheinberger's categories became productive, namely, literature studies. Mengaldo examines some of Georg C. Lichtenberg's notebooks from the period between 1789 and 1793, the so‐called *Sudelbücher*, as a site for private reflections on public objects, both technical and epistemic. The essay traces the emergence of *epistemic things* in these notebooks, and analyzes the place of thought experiments in the conceptual framework of *experimental systems*. More implicitly, but intriguingly, Mengaldo also draws out how writing is the technical practice par excellence for Rheinberger whereby thinking enters the *experimental system*.

### 2.3 Fragments

The third cluster of essays go under the heading of ***Fragments***. They discuss ways in which new approaches in the life sciences are fragmenting the world in distinct ways, and analyze the politics of writing history of science today.

**Soraya de Chadarevian** starts with an observation of the historical irony that while the response to the COVID‐19 pandemic saw high‐profile uses of molecular biological technologies, including mRNA vaccines, the history of molecular biology is increasingly told as one of hubris and has lost its appeal to historians. Yet, as de Chadarevian shows, this irony is an artefact. It is caused by restricting our focus to Crick's “central dogma,” or rather, an unjustifiably rigid interpretation of it. Rheinberger's periodization of molecular biology is presented as an alternative, and an expansion of perspectives on the early phase of molecular biology is proposed.

**Angela Creager**, in a way, continues this line of thought. The paper examines the rise of genetic explanations for various biological phenomena and argues that the ascendance of a narrative of genetic selfhood was reinforced by the central dogma and “fit well with liberal political thought, with its focus on the autonomous individual.” The analysis sheds light on the relations between mid‐twentieth century molecular biology and evolutionary biology but also sharpens Rheinberger's discussion of narrative and ideology in *Spalt und Fuge* through its exploration of the space between experiment and expectations.

**Ilana Löwy** then observes the role of expectations and ideology within promissory claims of genetic‐based approaches to precision medicine. She places recent incursions of genetic knowledge into the clinic, and the promises of contemporary pharmacogenetics, in historical and historiographical perspective, and she traces the long history of the rhetoric of hope that has regularly been employed whenever science and clinical practice met. Yet, Löwy also points to specific effects of the ongoing geneticization of the clinic, including “the possibility of re‐mining conserved biological materials for new information.”

**Jenny Bangham** highlights some implications of these new genetic meanings for historical work on human genetics. Taking her own archival experience as a point of departure, Bangham reports how she had to recalibrate her project to unravel the invisible labor of blood donors in the history of blood group research, because archival documents were reclassified as providing sensitive information, and thereby became unquotable. The essay interweaves reflections on the changing institutional politics of the Wellcome Trust, the impact of genetics to change what we consider as potentially troublesome information, and her own role as a historian. Having analyzed how blood group research conditioned so much work on human genetics, Bangham suddenly found herself confronted with human genetics conditioning what she could learn about the history of blood group research. The essay thereby echoes and enriches Rheinberger's call for attention to the politics of sources in the historiography of the life sciences.

The final contribution, co‐authored by **Esther Chen** and us, **the two editors**, also takes Rheinberger's politics of sources as a starting point, and juxtaposes the conceptual discussion of contemporary and outdated categories and the delicate distinction between sources and literature with the more practical considerations of a librarian. We are very grateful that Esther Chen, the MPIWG's librarian, agreed to have a conversation with us on the challenges of ordering knowledge in a digital age. Although the librarian vehemently denies it, the resulting arrangements and classifications can be profoundly influential and steer the historians’ attention from one set of questions and source materials to another. We finally agreed on the image of the contemporary library as a port where knowledge is ordered, exchanged, and distributed: an image that also captures the sense of displacement the academic worker sometimes feels in the contemporary library, no longer a destination but a packing station, no longer a resident but a passenger.

## 3 Epistemic Times

The collection ends with Hans‐Jörg Rheinberger's *Postscriptum*, which was the first manuscript submitted after the workshop to our journal. It delightfully explains, and to some extent justifies in retrospect, our choice of the three sections of this issue. However, in the age of post‐post‐postmodernism, and in any event within a Rheinbergian *Denkstil*, there is always ambivalence and fluidity. Principles of order may appear natural but they are inevitably contingent, and nobody can fully plan or predict the outcome of experimental endeavors like ours. After reading the submitted papers in their final form, we realized the multitude of alternative ways to pair and cluster essays that resonate with each other in compelling ways. There was, however, one recurrent theme that also was very much present at the workshop, namely the question of temporality and change beyond linear chronologies. The complexity of time and change came up in so many essays, across our carefully curated sections, that we decided to embrace the ambivalence, and rename the special collection to ***On Epistemic Times: Writing History 25 Years after Synthesizing Proteins in the Test Tube***.

The title reflects the particular engagement with epistemology and temporality in Hans‐Jörg Rheinberger's work—that we, of course, are not the first to notice^3^—by tentatively framing Rheinberger's heritage as a period of epistemic times. We suggest situating the book and the period by identifying four ways in which the collected essays highlight different types and layers of change—and various forms of borderline cases that can be read as transitions from one type of change to another:

1. *Changes within the life sciences over time*, focusing primarily on how new and old epistemological questions emerge in the study of the methods, objects, aims, and expectations of the life sciences of the twentieth and twenty‐first centuries.
2. *Changes within the historiography of life sciences*, illustrating in various ways how the focus of historiography has changed in the past years, putting particular emphasis on what has been written out of the history of science.
3. *Changes in our perception of historical epistemology*, providing initial steps towards a historicization of Rheinberger's approach towards historical epistemology.
4. *Changes in the way historical‐epistemological categories have been put to work*, demonstrating how topics, concepts, and methods of the history of *epistemic things* live on.

When using these layers of change as an alternative principle of order, a very different table of contents emerges, which we are suggesting (Table 1). By doing so, we also acknowledge the fact that most of our readers will not access these essays in their printed configuration, but in digital space, and in the order that they themselves find sensible.

**Table 1:**
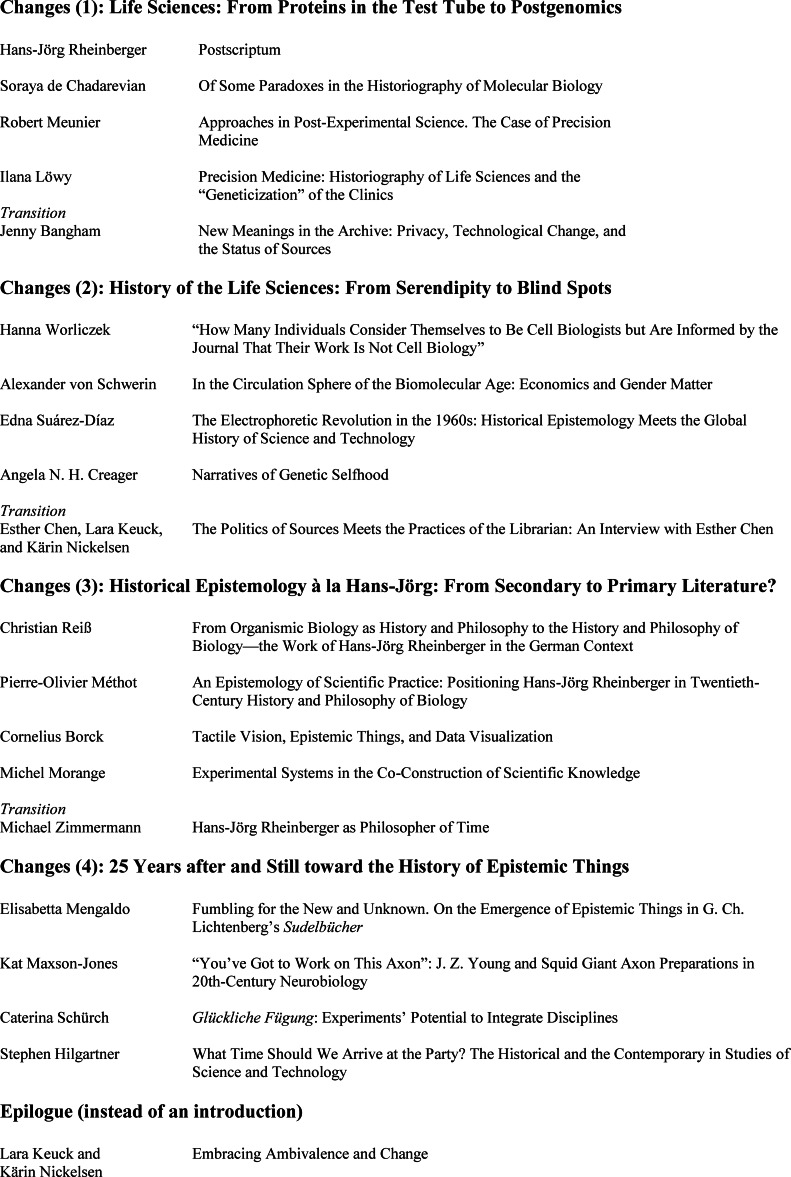
An alternative Table of Contents to this issue.

## 4 Endings

Many of the contributions in this volume offer tentative answers to the questions that sparked this project. They reflect on the meaning of writing the history of sciences now, the place of Rheinberger's historical epistemology in the contemporary landscape, and the changes in the field over the past 25 years. Yet they also raise more and equally pressing questions for the future. Beyond temporality and change, perhaps the most commonly shared contemplation is the crucial role of the scientific collective, which we have only just started to incorporate systematically into our epistemologies. We look forward to exploring this topic more thoroughly in future projects, collectively.

This special issue, just like so many of the histories it contains, is already the result of collective effort. It profited from the engagement of all authors during and after the workshop, and from the comments made by the additional discussants, Christina Brandt, Onur Erdur, Alfred Freeborn, Staffan Müller‐Wille, and Ohad Parnes. The essays not only went through peer‐review, but were also skillfully language‐edited and formatted by Aleksandra Ambrozy and Elizabeth Hughes. Henrik Hörmann, Klara Schwalbe, and Birgitta von Mallinckrodt of the Max Planck Research Group *Practices of Validation in the Biomedical Sciences* helped to organize the authors’ workshop. Dominik Knaupp from the *Berichte zur Wissenschaftsgeschichte* team in Munich as well as the WILEY team helped us to organize the many versions of the many contributions into this published form.

In this sense, our project has come to a conclusion. However, as Hilgartner's essay reminds us, collective projects, such as a party or an epistemological adventure, only come into existence through their performance. It is not preconfigured who and what makes the party, how it develops, and where it ends. Our own party, the joyful dance of sources and literature, categories and narratives in the historiography of the life sciences will certainly go on; and we will continue to struggle with the issues of time and change, both in our daily work and in our meta‐reflections. Linear temporality, as Rheinberger observed, is the easiest way out of this predicament, but also the least interesting form of history writing. We should therefore turn and face the strange—time may change us, but we can't trace time.^4^

## Footnotes

n1 See, e. g., Azzouni et al. 2011 and Santesmases et al. 2013a.

n2 For the short description of this paper's content (and some of the others further below), we are drawing on one of our reviewers’ astute summaries as source and inspiration, with this person's generous permission (thank you, Reviewer #1!). In a way, this is also a self‐reflective response to Worliczek's essay (in this volume) on the uses of reviewer comments.

n3 See, e. g., Santesmases et al. 2013b.

n4 We are indebted to David Bowie for this insight.
